# Consensus mutagenesis approach improves the thermal stability of system x_c_^−^ transporter, xCT, and enables cryo‐EM analyses

**DOI:** 10.1002/pro.3966

**Published:** 2020-11-11

**Authors:** Kazumasa Oda, Yongchan Lee, Pattama Wiriyasermkul, Yoko Tanaka, Mizuki Takemoto, Keitaro Yamashita, Shushi Nagamori, Tomohiro Nishizawa, Osamu Nureki

**Affiliations:** ^1^ Department of Biological Sciences Graduate School of Science, The University of Tokyo 7‐3‐1 Hongo, Bunkyo‐ku, Tokyo Japan; ^2^ Department of Structural Biology Max Planck Institute of Biophysics Frankfurt Germany; ^3^ Department of Collaborative Research for Bio‐Molecular Dynamics Nara Medical University Nara Japan

## Abstract

System x_c_^−^ is an amino acid antiporter that imports L‐cystine into cells and exports intracellular L‐glutamate, at a 1:1 ratio. As L‐cystine is an essential precursor for glutathione synthesis, system x_c_^−^ supports tumor cell growth through glutathione‐based oxidative stress resistance and is considered as a potential therapeutic target for cancer treatment. System x_c_^−^ consists of two subunits, the light chain subunit SLC7A11 (xCT) and the heavy chain subunit SLC3A2 (also known as CD98hc or 4F2hc), which are linked by a conserved disulfide bridge. Although the recent structures of another SLC7 member, L‐type amino acid transporter 1 (LAT1) in complex with CD98hc, have provided the structural basis toward understanding the amino acid transport mechanism, the detailed molecular mechanism of xCT remains unknown. To revealthe molecular mechanism, we performed single‐particle analyses of the xCT‐CD98hc complex. As wild‐type xCT‐CD98hc displayed poor stability and could not be purified to homogeneity, we applied a consensus mutagenesis approach to xCT. The consensus mutated construct exhibited increased stability as compared to the wild‐type, and enabled the cryoelectron microscopy (cryo‐EM) map to be obtained at 6.2 Å resolution by single‐particle analysis. The cryo‐EM map revealed sufficient electron density to assign secondary structures. In the xCT structure, the hash and arm domains are well resolved, whereas the bundle domain shows some flexibility. CD98hc is positioned next to the xCT transmembrane domain. This study provides the structural basis of xCT, and our consensus‐based strategy could represent a good choice toward solving unstable protein structures.

## 1 INTRODUCTION

Amino acid transporters play critical roles in humans: the genome encodes at least 66 known transporter members that are classified into 11 discrete solute carrier (SLC) families.^1^ Among them, the heteromeric amino acid transporters (HATs) are unique members composed of two subunits, the light chain (SLC7) and the heavy chain (SLC3), linked by a conserved disulfide bridge. The light chain consists of 12 transmembrane helices and belongs to the amino acid‐polyamine‐organocation transporter (APC) superfamily. The heavy chain has a large extracellular domain with a single transmembrane helix, and facilitates trafficking of the light chain^6^. The human genome encodes eight SLC7 members (SLC7A5–11, 13), and each specifically associates with either SLC3A1 or SLC3A2.^2^, ^3^

The system x_c_^−^ amino‐acid transporter (SLC7A11, also named xCT) is a Na^+^‐independent electroneutral exchange system for cystine/glutamate.^4^ This transport system generally uptakes extracellular cystine in exchange for intracellular glutamate, at a 1:1 ratio.^5^ Cystine, the oxidized dimer form of cysteine, is the predominant form of cysteine in plasma^6^ and required for the synthesis of intracellular glutathione (GSH), which plays a principal role in cellular redox homeostasis.^7^ Previous studies have shown that many cancer cell lines highly express xCT, resulting in an elevated intracellular concentration of GSH, which mitigates oxidation or electrophilic attack by reactive oxygen species (ROS).^5^, ^8^, ^9^ These cancer cell lines become resistant to oxidative stress, and thus xCT blockers work as tumor growth inhibitors. Notably, sulfasalazine, a drug commonly prescribed for ulcerative colitis or rheumatoid arthritis, was recently found to act as a specific inhibitor of xCT‐mediated cystine transport and is now undergoing clinical trials for mono‐ and combination therapies against different types of cancer.^10^

Although xCT is biologically important and could be a major candidate for cancer treatment and anticancer drug development, its detailed molecular mechanism remains unclear. Recent cryo‐EM structures of LAT1‐CD98hc and LAT2‐CD98hc revealed the overall architecture of the HATs and indicated the interactions between the light and heavy chains.^11^, ^12^, ^13^ However, owing to the low sequence similarity between xCT and LAT1 (45.69% identity, whereas all human HATs share only 30–63% identities), it is difficult to deduce the mechanisms of cystine/glutamate recognition and exchange by xCT and its inhibition by sulfasalazine, on the basis of the LAT1 structure or other prokaryotic SLC7 homologs. In addition, among HATs, xCT has the unique interaction partner CD44v, a splice variant of a hyaluronan receptor that has been proposed to interact with and stabilize xCT. CD44+ cancer cells could thereby regulate the intracellular level of GSH through xCT, resulting in improved growth.

In this study, towards the goal of elucidating the mechanism of system x_c_^−^, we used a consensus‐based mutagenesis approach, which improved the structural stability of xCT‐CD98hc and enabled the purification and cryo‐EM analyses of the complex. We now report the 6.2 Å resolution map of the consensus‐based xCT‐CD98hc, which reveals the overall architecture of system x_c_^−^ at the secondary structure level and serves as a basis for further biochemical and structural characterizations of this complex.

## 2 MATERIALS AND METHODS

### 2.1 *Cloning and expression*

xCT‐CD98hc complexes were expressed as previously described.^11^ Briefly, the sequences encoding full‐length human xCT (SLC7A11; UniPlot ID Q9UPY5) and the CD98hc isoform *c* (SLC3A2 isoform 1; UniProt ID P08195‐1) were amplified from human universal reference cDNA (Zyagen) and cloned individually into the pEG BacMam vector. xCT was fused with a C‐terminal FLAG epitope, and CD98hc was fused with an N‐terminal His8 tag and enhanced green fluorescent protein (eGFP), followed by the tobacco etch mosaic virus (TEV) protease cleavage site. Baculoviruses were generated in *Spodoptera frugiperda* Sf9 cells using the Bac‐to‐Bac system (Invitrogen). HEK293S GnTI− cells were used for expression.

### 2.2 *Purification and consensus‐based construct design*

**FIGURE 1:**
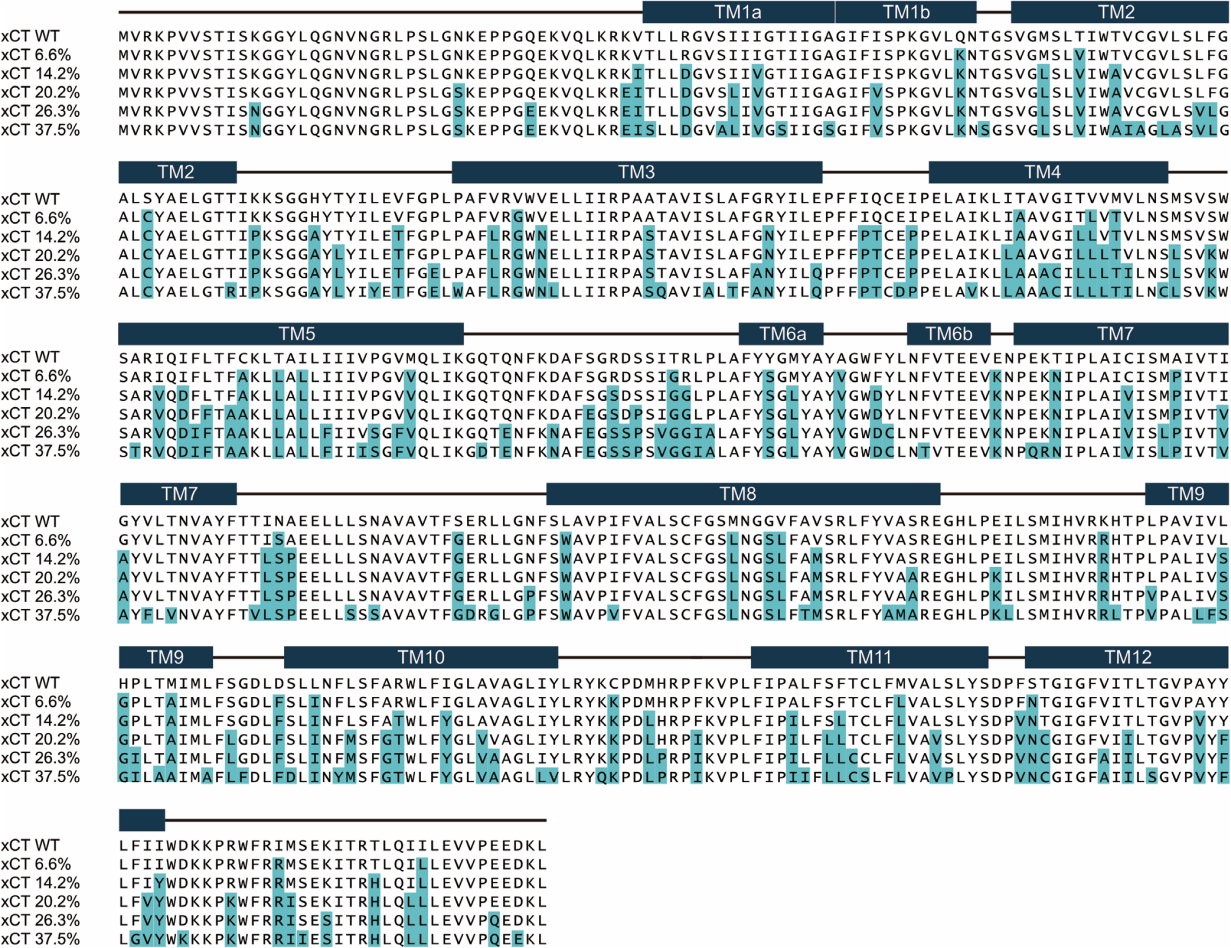
Sequence alignment of xCT wild type and consensus constructs. Amino acid alignment of the wild‐type and consensus‐mutated xCT sequences. The numbers after xCT indicate the ratios of mutated residues in individual constructs, as compared to the wild type. Secondary structures of xCTcons are indicated above the sequences. The teal background color indicates consensus mutations

We first tried to purify the wild‐type xCT‐CD98 complex, according to the previously reported purification method for LAT1‐CD98hc.^11^ Briefly, the complex was solubilized in DDM and CHS (Figure S1), and then purified by anti‐FLAG M2 affinity chromatography and a GFP‐nanotrap in digitonin detergent. After digestion with TEV protease, we planned to concentrate the xCT‐CD98hc complex for further purification by size‐exclusion chromatography. However, we failed to purify the xCT‐CD98hc complex. Wild‐type xCT is extremely unstable and the concentrated xCT tends to aggregate (Figure S2). To improve the stability of the xCT‐CD98hc complex, we applied a consensus mutagenesis approach to the xCT transmembrane regions.^14^ This approach introduces the most prevalent amino acid at a given position, based on the multiple sequence alignment of homologous proteins from many species. Previous reports have experimentally confirmed that this approach improves the thermostability of immunoglobulin domains,^15^ GroEL minichaperones,^16^ and the WW domain.^17^ In addition, more recent reports have described the application of consensus mutagenesis to membrane proteins for X‐ray crystallography.^18^, ^19^ To reliably predict stabilizing mutations, we calculated the ratio of amino acid frequencies^15^ f (cons) / f (WT) for each position, where f (cons) and f (WT) are the frequencies of the most frequent amino acid in a given column and that of the wild‐type, respectively (https://github.com/TaizoAyase/consensus_creator). We then prepared several constructs combining all mutations that have higher f (cons) / f (WT) than a specified threshold (Y.L. et al., H.F. et al., manuscripts in preparation). Thus, we obtained a panel of xCT constructs with different numbers of consensus mutations (Figure 1).

**FIGURE 2:**
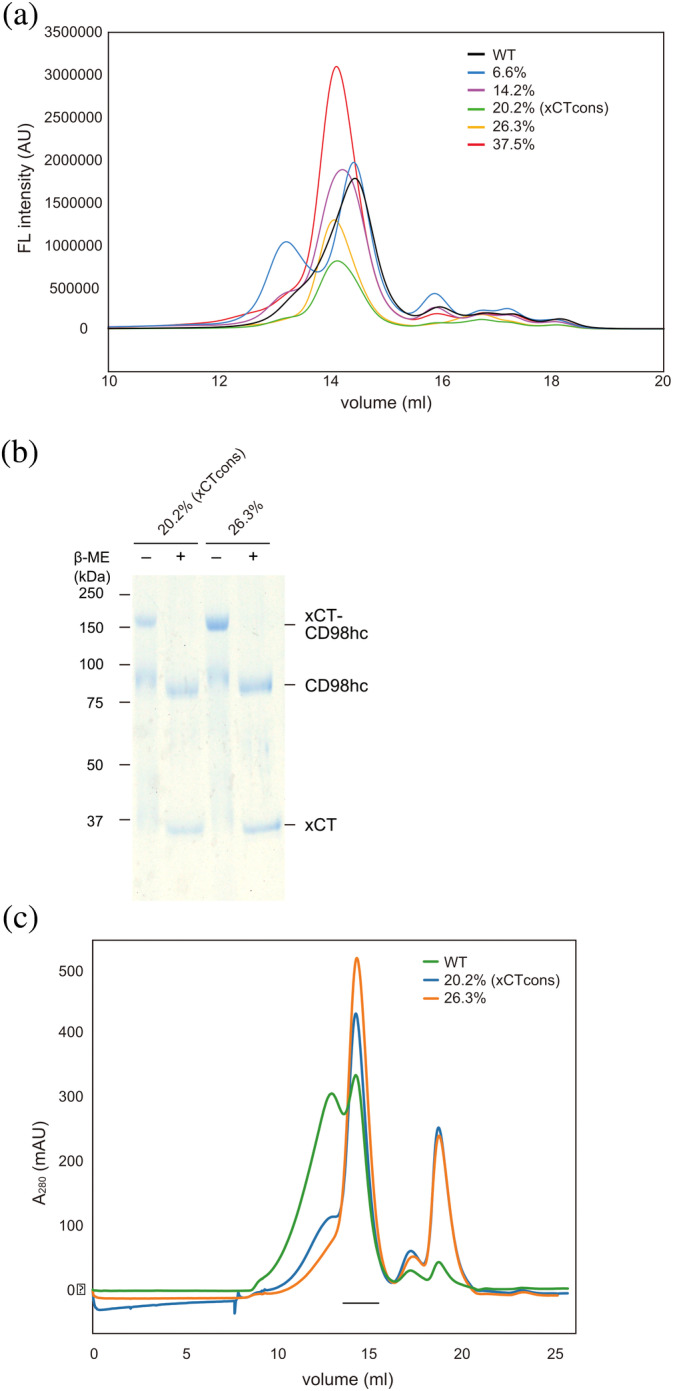
Purification of xCT consensus constructs. (a) FSEC profiles of GFP‐fused xCT wild type and consensus mutated constructs, detected by GFP fluorescence. (b) SDS‐PAGE analyses of the two consensus mutated constructs, with or without 20 mM β‐ME. (c) Size‐exclusion chromatography profile of the xCT‐CD98hc complex WT (green) and two consensus mutated constructs (blue and orange). The fractions used for structural and functional analyses are indicated by the black line

The expression screening of these mutated xCT constructs was performed by fluorescence‐detection size‐exclusion chromatography (FSEC) and FSEC thermostability assays (FSEC‐TS) [Figure 2(a), S3]. Based on the FSEC and FSEC‐TS results, we identified two constructs (14.2% and 20.2%) as suitable candidates and purified these constructs. The SDS‐PAGE analysis indicated that these two constructs stably formed the heterodimeric complex with CD98hc [Figure 2(b)], and the size exclusion chromatography showed single peaks of the two purified complexes, as compared to the double peaks of WT [Figure 2(c)]. The purified complexes were concentrated to 4.5 mg/mL and subjected to cryo‐EM single‐particle analyses. The complex with f (cons) / f (WT) = 2.22 showed better behavior, and is hereafter designated as xCTcons. The xCTcons protein has 101 amino acid substitutions (20.2% of total amino acids) as compared to wild‐type xCT (Figure 1).

### 2.3 *Cryo‐electron microscopy data collection*

The xCTcons‐CD98hc complex (3 μL at 4.5 mg/mL) was applied onto a glow‐discharged Quantifoil holey carbon grid (R1.2/1.3, Cu/Rh, 300 mesh), blotted for 10 s at 6°C, Force level 4 in 100% humidity, and plunge‐frozen in liquid ethane by using a Vitrobot Mark IV. Initial cryo‐EM screening was performed on a 200 kV Talos Arctica electron microscope (Thermo Fisher Scientific), equipped with a GIF Quantum energy filter (Gatan) and a K2 direct electron detector (Thermo Fisher Scientific) in the electron counting mode. High‐resolution cryo‐EM imaging of the xCT‐CD98hc complex was performed with a 300 kV Titan Krios G3i electron microscope (Thermo Fisher Scientific), equipped with a GIF Quantum energy filter (Gatan) and a K3 direct electron detector (Thermo Fisher Scientific) in the electron counting mode. Imaging was performed at a nominal magnification of ×96,000, corresponding to a calibrated pixel size of 0.8346 Å per pixel (The University of Tokyo). Each movie was recorded for 3.65 s and subdivided into 40 frames. The electron flux rate was set to 13.5 e − per pixel per second at the detector, resulting in an accumulated exposure of 49.76 e−/Å^2^ at the specimen. The data were automatically acquired by the image shift method using the SerialEM software,^20^ with a defocus range of −0.8 to −1.6 μm. We recorded 6,156 micrographs of the purified xCTcons‐CD98hc complex with the 300 kV Titan Krios microscope.

### 2.4 *Image processing*

For all datasets, the dose‐fractionated movies were subjected to beam‐induced motion correction, using MotionCor2^21^ or RELION‐3,^22^ and the contrast transfer function (CTF) parameters were estimated using CTFFIND4.1.13.^23^ Initially, 1,469 particles were picked from the 9 micrographs by using the Laplacian‐of‐Gaussian picking function in RELION‐3^22^ and extracted with down‐sampling to a pixel size of 2.95 Å per pixel. These particles were subjected to several rounds of 2D classification, and the best class was used for the reference in the next step. Using the reference‐based picking function of RELION‐3, 1,340,640 particles were picked from all micrographs [Figure S4(a), S4(b)]. These particles were subjected to several rounds of 2D and 3D classifications [Figure S4(b), S4(c)]. The best class contained 86,395 particles, which were then re‐extracted with a pixel size of 1.48 Å per pixel and subjected to 3D refinement. The final 3D refinement and postprocessing yielded a map with a global resolution of 6.18 Å, according to the FSC = 0.143 criterion^24^ [Figure S4(d‐f)].

### 2.5 *Model building and structure refinement*

Based on the LAT1‐CD98hc complex structure (PDB: 6RIS), a homology model for the xCTcons transmembrane domain was built using the Phyre2 server,^25^ where 454 residues were modeled with 100.0% confidence by the single highest scoring template. We fitted this homology model and a complete model of CD98hc, from the LAT1‐CD98hc complex structure, into the density map by rigid‐body fitting with UCSF Chimera.^26^ After the fitting, the xCTcons‐CD98hc complex model was relaxed into the density with Rosetta and refined with its asymmetric refinement procedure.^27^ The density weight parameter was 10, and we used the multiple cycles strategy. Based on the MolProbity score^28^ and the average model‐map FSC, the top‐scored structure was selected and used for model deposition and figure presentation. The Ramachandran plot showed 90.51% of the residues in the favored regions and 7.31% in the allowed regions. Figures were prepared with Cuemol (http://www.cuemol.org/) or ChimeraX (https://www.rbvi.ucsf.edu/chimerax/).

### 2.6 *Preparation of the xCTcons‐CD98hc‐Fab complex*

In the previous LAT1‐CD98hc report,^11^ commercial anti‐CD98 antibodies were used to increase the particle size and add features for image alignment. We also prepared the xCTcons‐CD98hc‐Fab complex based on this method, using clone MEM‐108 as the antibody, and the final concentration of the xCTcons‐CD98hc‐Fab complex was ~8.5 mg/mL.

### 2.7 *Transport assay in transfected cell lines*

The transport activities of the wild‐type xCT and xCTcons constructs were measured in HEK293 cells. The cells were seeded on poly‐d‐lysine‐coated 24‐well plates at 2 x 10^5^ cells/well and cultured in DMEM containing 10% FBS, at 37°C and 5% CO_2_. At 24 h after seeding, plasmids encoding CD98hc and wild‐type xCT (or xCTcons) were transfected into the cells at a 1:1 molar ratio, using Lipofectamine 3000 (Thermo) according to the manufacturer’s protocol. As a negative control, the cells were transfected with the empty vector instead of the xCT constructs. The transfected cells were continuously cultured for 2 days. The transport assay was performed as described previously, with some modifications.^29^ Briefly, the transport rates of 50 μM radioisotope‐labeled substrate (l‐[^14^C]‐Glutamic acid (4 Ci/mol; Moravek Biochemicals)) were measured for 2 min in Na^+^‐free HBSS, pH 7.4. After terminating the reaction and lysing the cells, an aliquot of the lysate was used to measure the protein concentration by a BCA protein assay (Takara Bio). The lysate was mixed with Optiphase Hisafe 3 (PerkinElmer), and the radioisotope activity was measured with an LSC‐8000 β‐scintillation counter (Hitachi).

Expression of the xCT constructs was determined by western blotting. The transfected cells were collected and disrupted with lysis buffer [20 mM Tris–HCl, pH 7.4, 150 mM NaCl, 10% v/v glycerol, 1% w/v FC‐12 (Anatrace) and protease inhibitor cocktail (Nacalai)] for 30 min at 4°C, and then centrifuged at 20,000 xg for 30 min. The cell lysates (180 μg) were subjected to western blotting, as described.^29^ Signals of the xCT constructs were detected via the immunoreaction to an anti‐FLAG antibody (HRP‐conjugated; Sigma). The signals were developed with the Immobilon Forte Western HRP substrate (Millipore), and the images were detected by the ChemiDoc Touch Imaging System (Bio‐Rad).

## 3 RESULTS AND DISCUSSION

**FIGURE 3:**
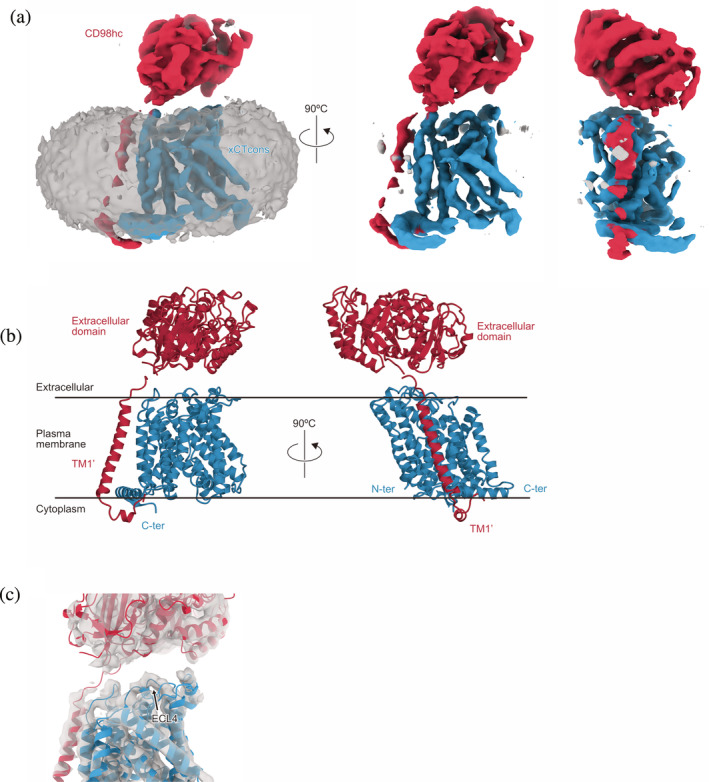
Overall structure of the xCTcons‐CD98hc complex. (a) Cryo‐EM map of the xCTcons‐CD98hc complex. Map segments are colored according to the subunits. The blue segment represents xCTcons, the red segment represents CD98hc, and the grey segment represents the micelle. (b) Cartoon model of xCTcons‐CD98hc, with xCTcons (blue) and CD98hc (red). (c) Structural model of the xCTcons‐CD98hc complex in the cryo‐EM density map. The grey portion represents the cryo‐EM map. xCTcons (blue) and CD98hc (red) are shown as cartoon models

Consistent with the previously obtained cryo‐EM 3D‐maps of LAT1‐CD98hc and LAT2‐CD98hc,^11^, ^12^, ^13^ the cryo‐EM 3D‐map of the xCTcons‐CD98hc complex contained two differently sized densities [Figure 3(a)]. Based on the previous cryo‐EM structure, the smaller density corresponds to the CD98hc extracellular domain and the larger density represents the micelle‐embedded xCTcons transmembrane domain and the N‐terminal cytoplasmic domain of CD98hc. When viewed from the extracellular side, the CD98hc‐ED density is laterally displaced by 20 Å from the center of the TMD‐density. A similar displacement was also observed in the previous cryo‐EM 3D maps. Its orientation is in good agreement with the previous crosslinking data, suggesting a similar overall subunit arrangement for xCTcons‐CD98hc.

The 6.2 Å resolution cryo‐EM map, according to the FSC = 0.143 criteria, resolved the secondary structural features of xCTcons‐CD98hc to allow the protein model fitting into the density. We created a homology model of the xCTcons‐transmembrane domain based on the previous LAT1‐CD98hc structure by the structure prediction server Phyre2,^25^ and manually fitted the transmembrane domain model and the atomic model of CD98hc into the density by rigid‐body fitting. The model was subsequently relaxed into the density with Rosetta^27^ [Figure 3(b)]. The N‐terminal transmembrane domain of CD98hc is adjacent to the TMD of xCTcons, consistent with the previous cryo‐EM model. The cryo‐EM density is better resolved at the contact sites between CD98hc and xCTcons, especially extracellular loop 4 (ECL4) [Figure 3(c)], as compared to the other parts, in which some transmembrane alpha helices are not separated and appear as partially connected densities.

**FIGURE 4:**
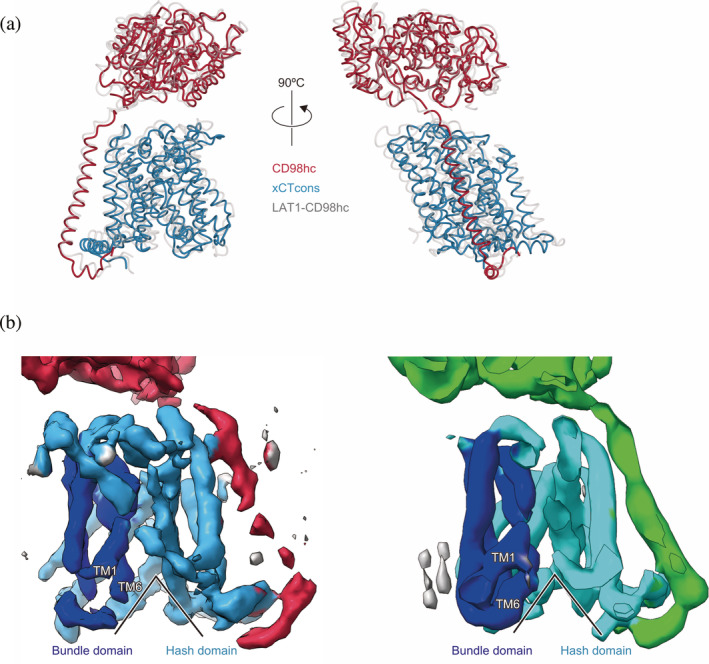
Structural and cryo‐EM map comparisons with the LAT1‐CD98hc complex. (a) Structural comparison with the LAT1‐CD98hc complex. The xCTcons and CD98hc proteins are colored as in Figure 3. The LAT1‐CD98hc complex is colored grey. (b) Cryo‐EM map comparison with the LAT1‐CD98hc complex. The left map represents xCTcons‐CD98hc and the right map represents the 7.0 Å low‐pass filtered LAT1‐CD98hc complex. In the maps, the bundle domain is blue, the hash domain is cyan, and CD98hc is red or green. The black line indicates that both structures are in the inward‐facing conformations

To further analyze the structures of xCTcons and LAT1, we compared the current map of xCTcons‐CD98hc with the map of LAT1‐CD98hc low‐pass‐filtered at a similar resolution (7.0 Å) (Figure 4). TM1 and TM6 are apart from the substrate translocation pathway and create the cytoplasmic open cavity, similar to those in LAT1 structures, indicating that our structure captures an inward‐open state of xCTcons. The substrate‐binding pocket is also located at the same position as that of LAT1 and is empty, consistent with the absence of a substrate in our sample preparation.

**FIGURE 5:**
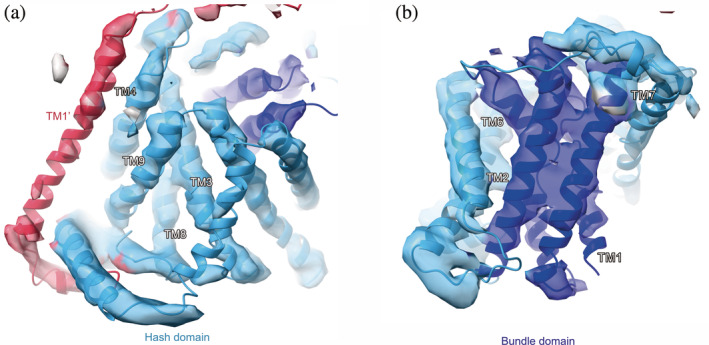
Structures of the hash and bundle domains of the xCTcons‐CD98hc complex in the cryo‐EM map. (a) Structure of the hash domain of the xCTcons‐CD98hc complex in the cryo‐EM map. The sky‐blue segment represents the cryo‐EM map around the hash domain. (b) Structure of the bundle domain of the xCTcons‐CD98hc complex in the cryo‐EM map. The blue segment represents the cryo‐EM map around the bundle domain

The ten TMs (1, 2, 3, 4, 5, 6, 7, 8, 9, 10) represent the common architecture of the APC superfamily consisting of three subdomains, hash, bundle, and arms. The cryo‐EM densities of the hash domain, characterized by the four helices arranged as a ‘hash’ character, are clearly resolved [Figure 5(a)]. By contrast, the cryo‐EM densities of the bundle domain have lower resolution, and the four‐helices in the bundle are partially connected together [Figure 5(b)]. During the transport cycle, this domain is considered to move as a dynamic gate and expose the central substrate pocket, alternating between the two sides of the membrane. The indistinct electron density suggests the structural flexibility of this domain. Notably, the current cryo‐EM density clearly showed the existence of a C‐terminal helix running parallel to the membrane, which was not visible in our previous structure of LAT1‐CD98hc, but detected in another study [Figure 3(a), 3(b)]. We speculate that the partial destabilization or disruption of the LAT1‐CD98hc complex structure was caused by delipidation during the purification procedure, in our previous preparation of LAT1‐CD98hc.

To improve the resolution of the xCTcons‐CD98hc complex, we also recorded cryo‐EM images of the xCTcons‐CD98hc complex with an anti‐CD98hc Fab fragment, which increases the particle size and adds features for imaging alignment. We used the commercial anti‐CD98hc antibody, clone MEM‐108, as in the previous study of the LAT1‐CD98h structure.^11^ We recorded 958 micrographs of the xCT‐CD98hc‐MEM108 Fab complex with a 300 kV Titan Krios microscope (Figure S5). However, the cryo‐EM density and the resolution were not improved, suggesting that the resolution is limited by the intrinsic flexibility of the protein, rather than the small molecular size or lack of structural features.

Here we have shown that the consensus‐based mutations improved the stability of xCT and enabled the production of the cryo‐EM 3D reconstruction map, which was sufficient to model the secondary structures. This approach could be effective for structural studies of other flexible proteins, but may possibly affect their functions. In the current case, xCTcons contains mutations of 20.2% residues over the whole protein, including the putative substrate binding pocket (Figure S6). We performed functional analyses of wild‐type and consensus mutated xCT, by using the radiolabeled L‐[^14^C]‐Glutamate uptake assay into HEK293 cells to monitor the effects of the mutations. Although wild‐type xCT shows Na^+^‐independent glutamate transport activity, xCTcons has lost this activity (Figure S7). These results suggest that at least one of the consensus mutations altered the substrate specificities or abolished the amino acid transport activity. These results were within our expectations, since some mutations are located near the substrate‐binding pocket. Further work will be required to obtain a high‐resolution structure of xCT for the functional characterization.

Despite the recent technological advances in cryo‐EM, the sample preparation still remains as a major bottleneck for high‐resolution structure determination. Especially, most human membrane proteins are unstable and often difficult to express or purify. Previous protein evolution studies suggested that some mutations providing a new function could be destabilizing.^30^, ^31^, ^32^ The sequence statistics, such as the consensus mutagenesis approach, might provide a way to change these moderately destabilizing mutations to the optimal residues. Along with newly developed detergents, amphiphilic polymers, and fast automated vitrification systems,^33^ the consensus mutagenesis approach will allow us to solve the structures of unstable or challenging proteins.

## AUTHOR CONTRIBUTIONS

**Kazumasa Oda:** Data curation; formal analysis; investigation; methodology; writing‐original draft. **Lee Yongchan:** Data curation; investigation; writing‐review and editing. **Pattama Wiriyasermkul:** Data curation. **Yoko Tanaka:** Data curation. **Mizuki Takemoto:** Investigation; software; validation. **Keitaro Yamashita:** Conceptualization; data curation; investigation; writing‐review and editing. **Shushi Nagamori:** Data curation. **Tomohiro Nishizawa:** Data curation; investigation; writing‐original draft. **Osamu Nureki:** Conceptualization; funding acquisition; project administration; supervision; writing‐original draft.

## Supporting information


**Figure S1** Supporting information
