# Supplementary figures and images for: Consensus mutagenesis approach improves the thermal stability of system xc − transporter, xCT, and enables cryo‐EM analyses
Source: Protein Sci. 2020 Nov 11;29(12):2398–407. doi: 10.1002/pro.3966 (PMC7679960; doi:10.1002/pro.3966)

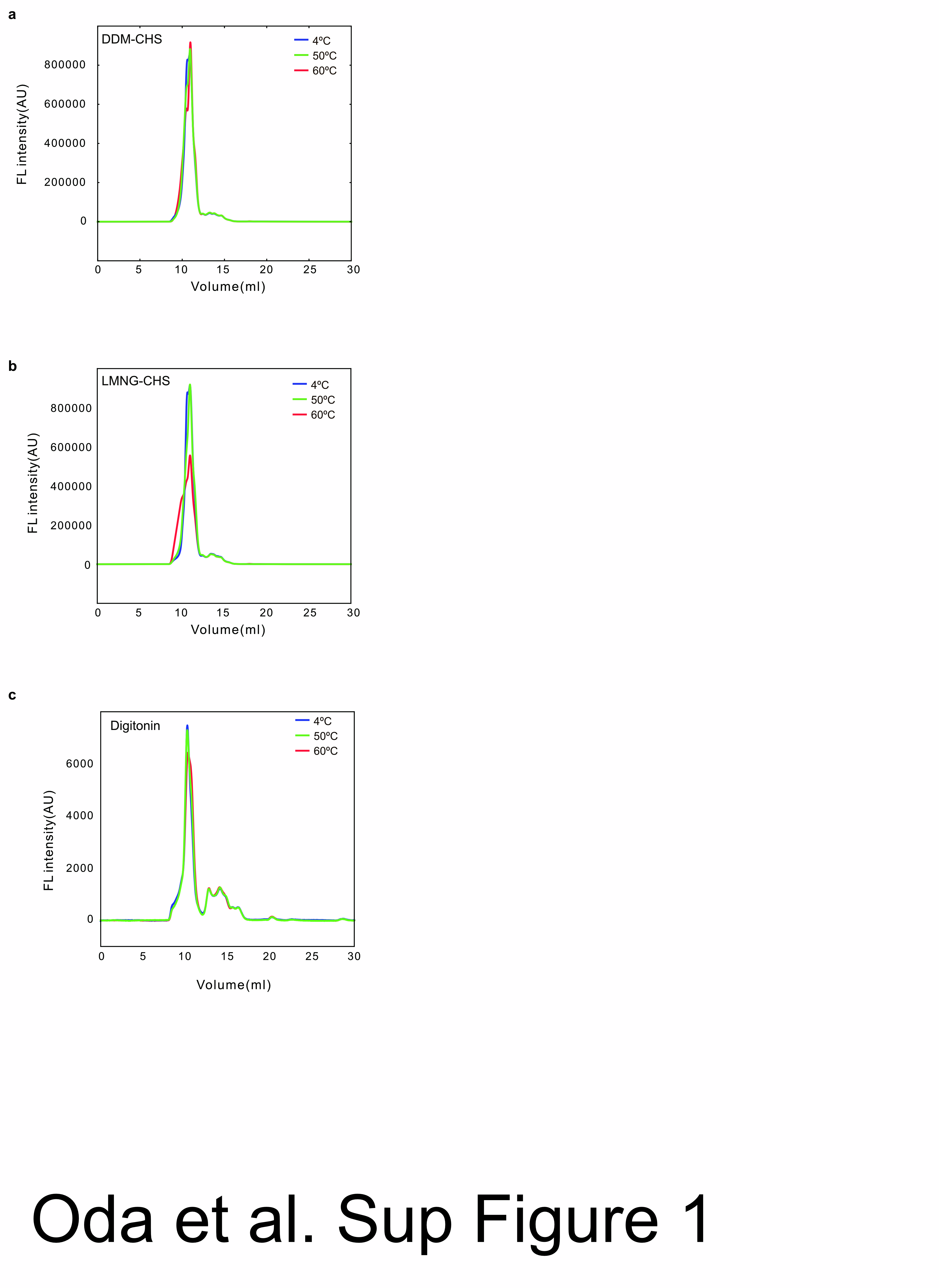

Supplement: Supplementary file 1 — Figure S1 Supporting information [file PRO-29-2398-s001.zip › pro3966-sup-0001-Fig1.tiff]

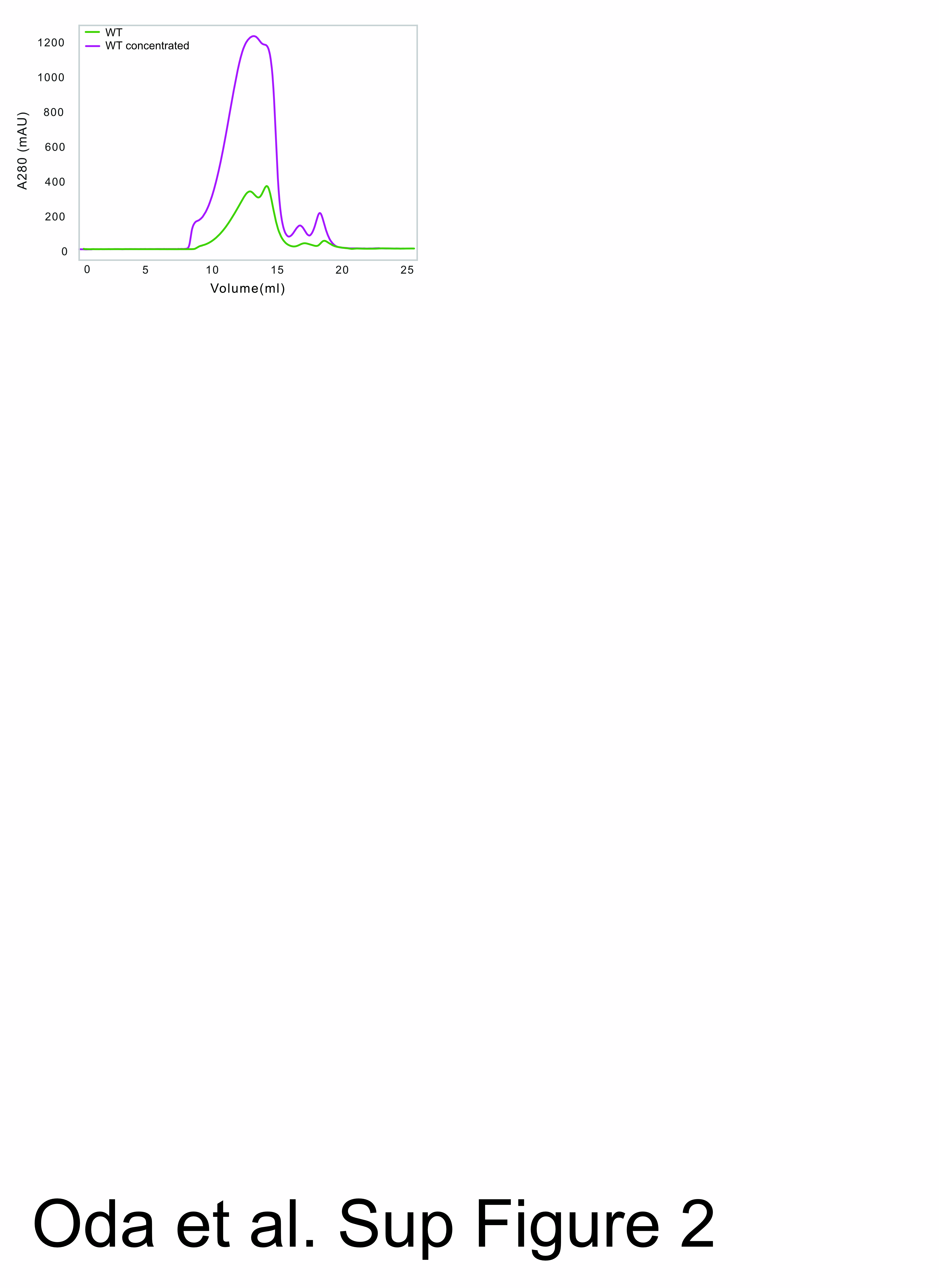

Supplement: Supplementary file 1 — Figure S1 Supporting information [file PRO-29-2398-s001.zip › pro3966-sup-0002-Fig2.tiff]

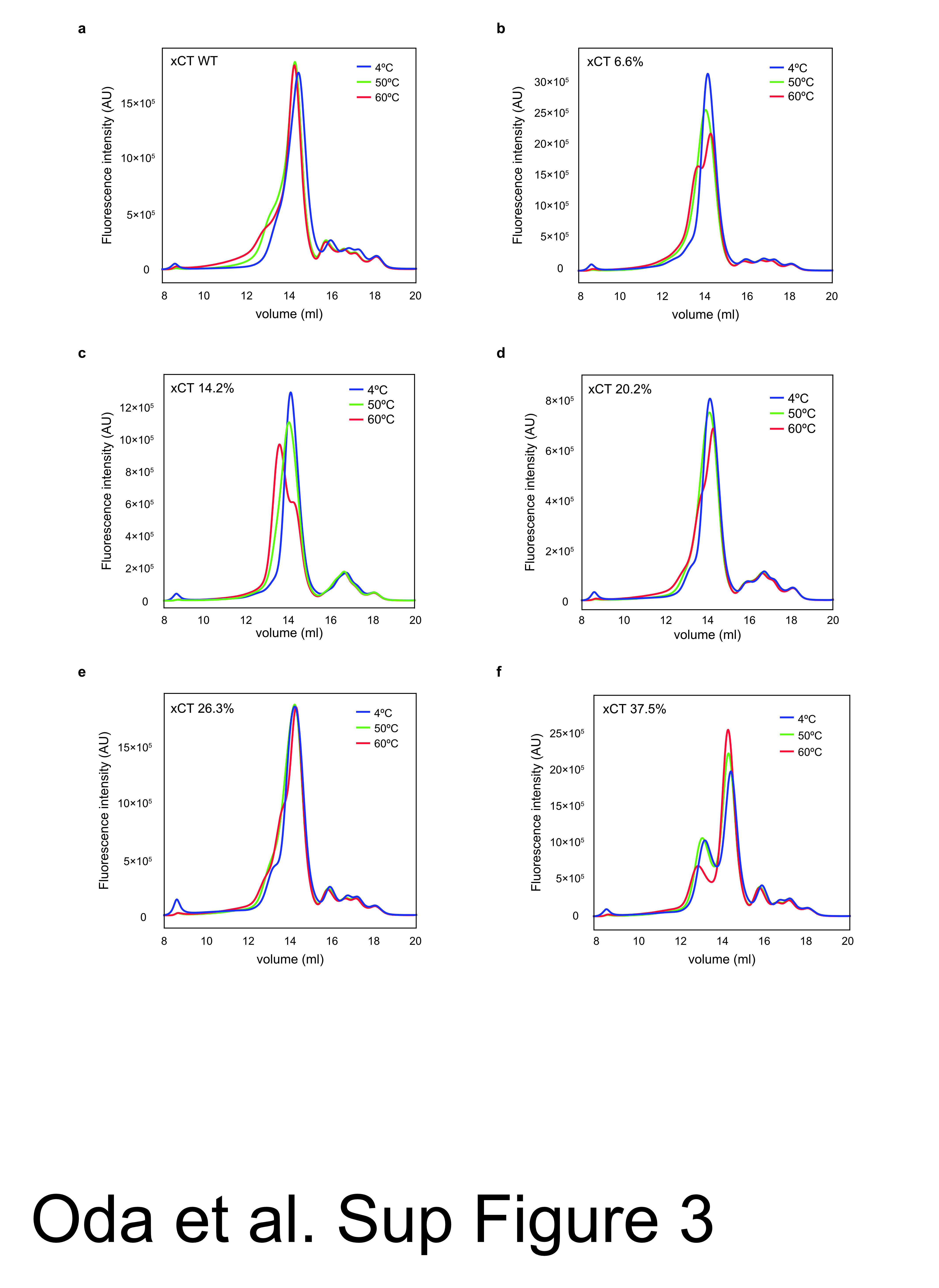

Supplement: Supplementary file 1 — Figure S1 Supporting information [file PRO-29-2398-s001.zip › pro3966-sup-0003-Fig3.tiff]

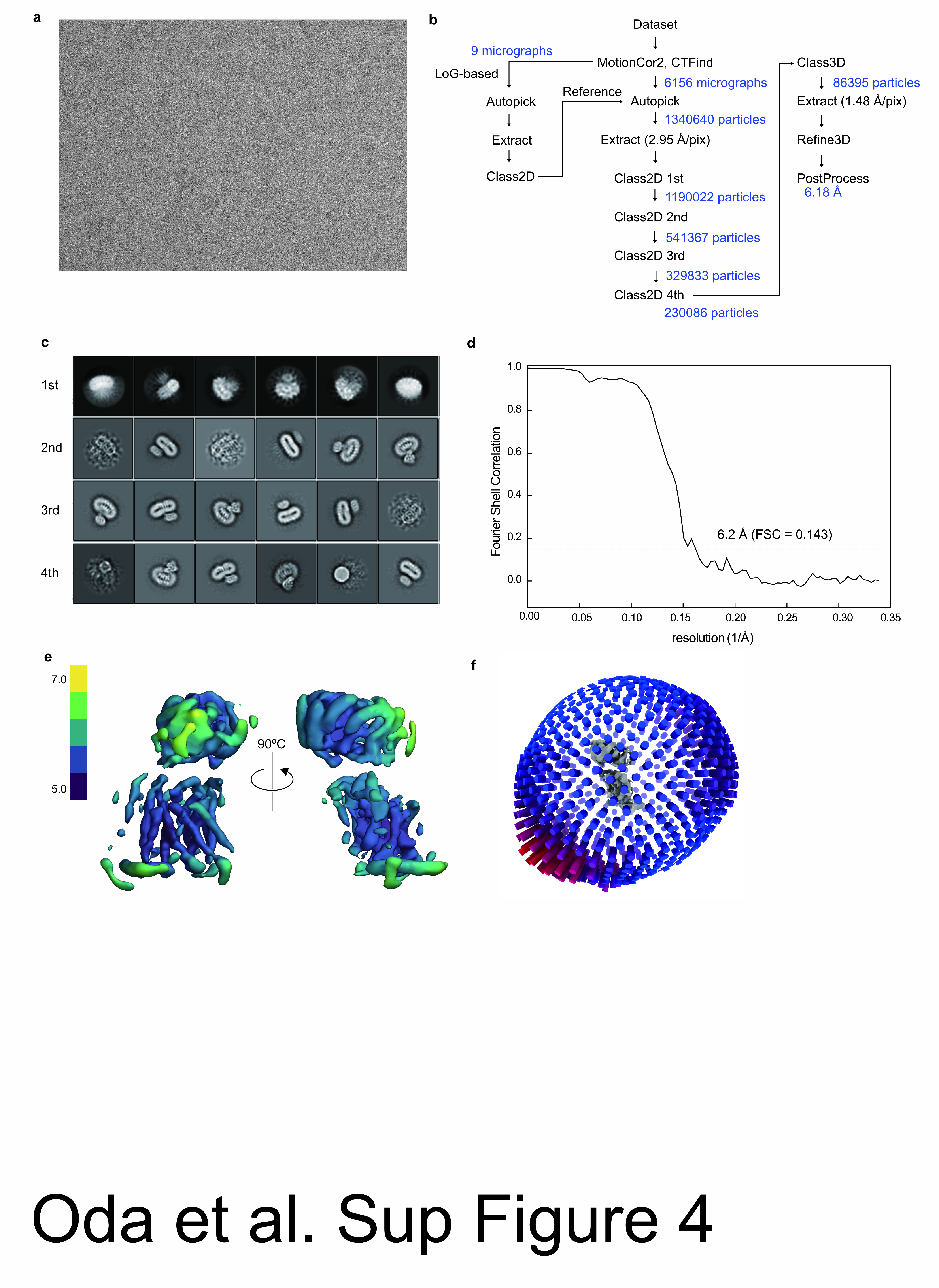

Supplement: Supplementary file 1 — Figure S1 Supporting information [file PRO-29-2398-s001.zip › pro3966-sup-0004-Fig4.tiff]

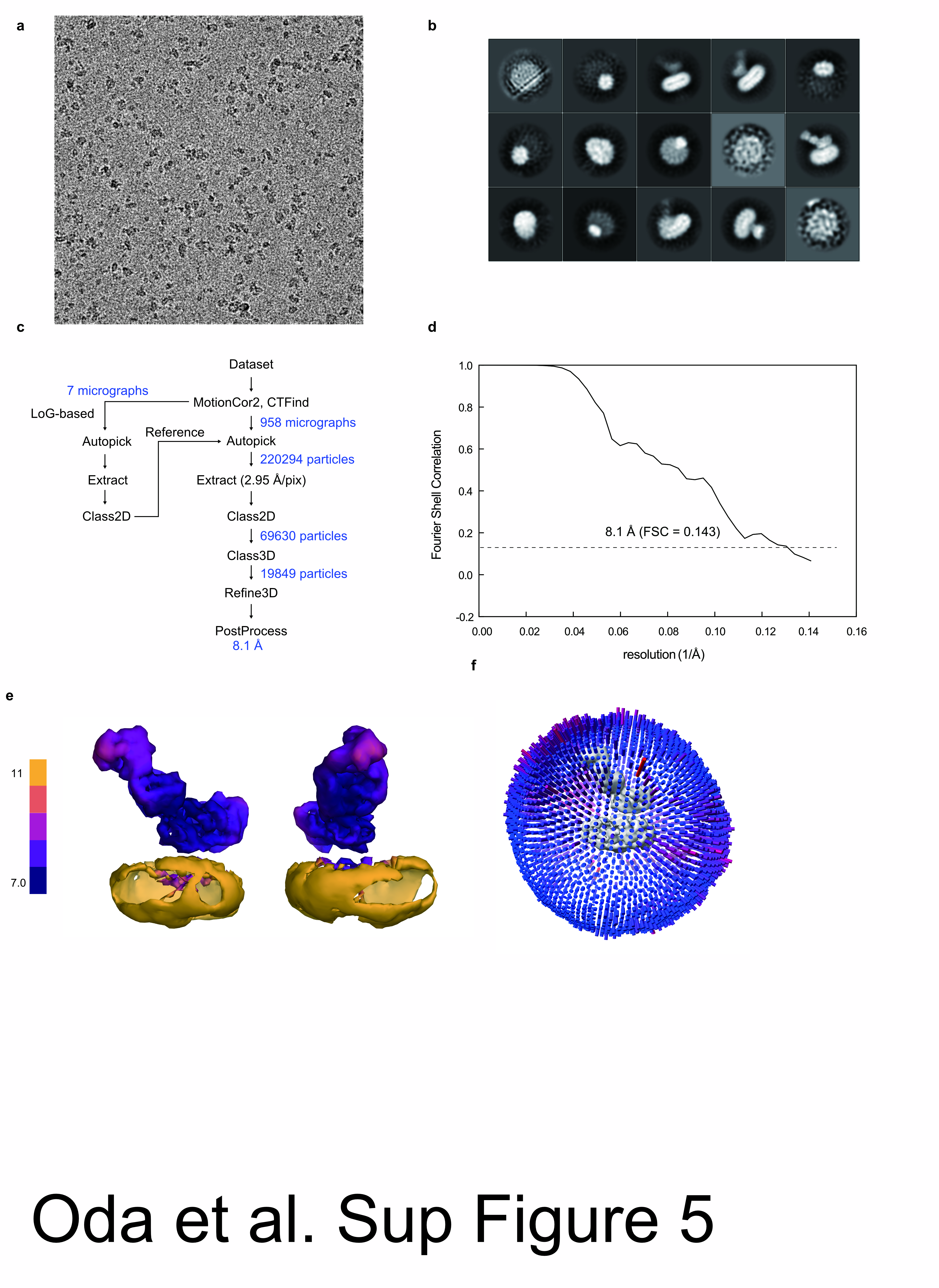

Supplement: Supplementary file 1 — Figure S1 Supporting information [file PRO-29-2398-s001.zip › pro3966-sup-0005-Fig5.tiff]

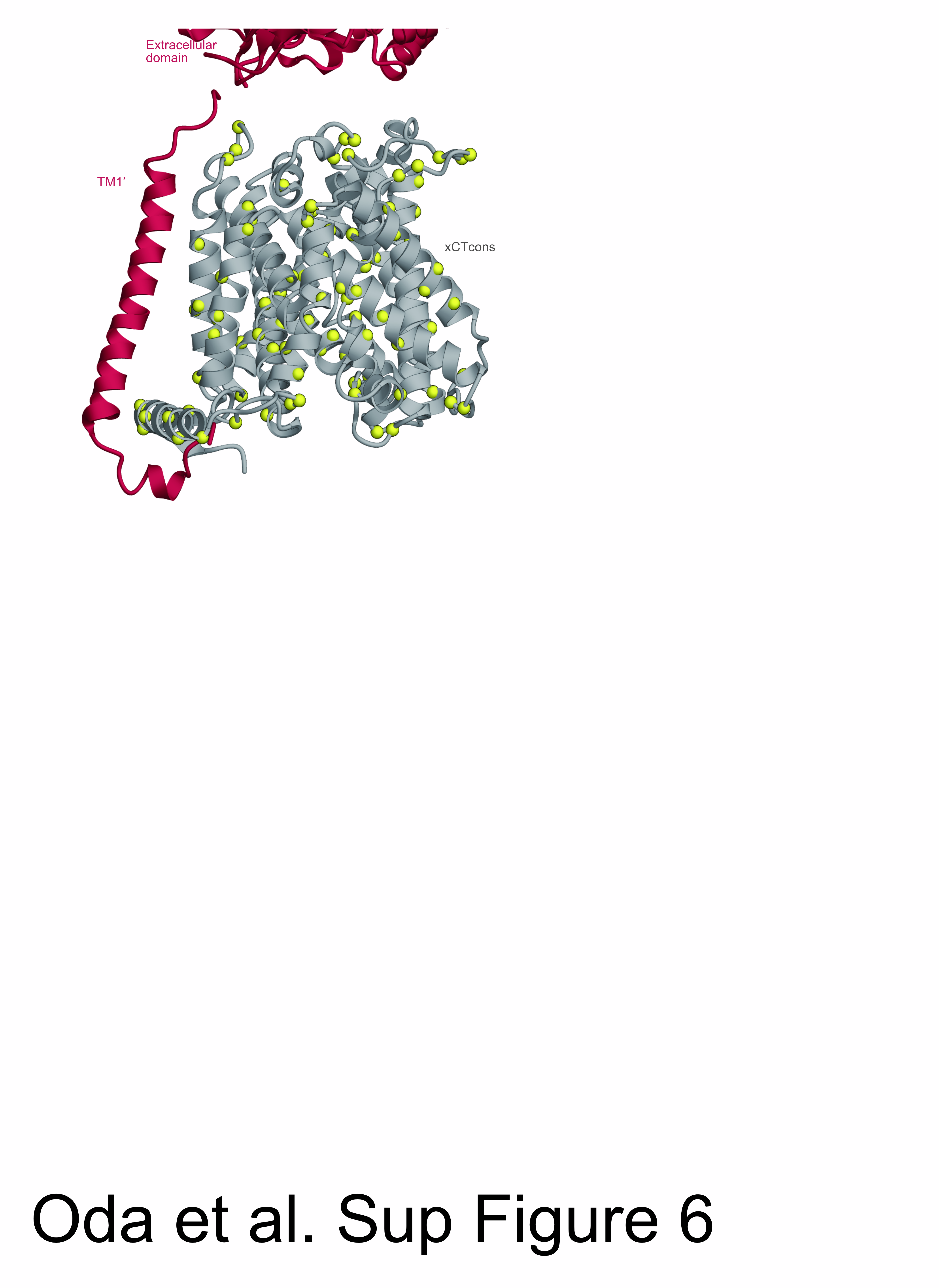

Supplement: Supplementary file 1 — Figure S1 Supporting information [file PRO-29-2398-s001.zip › pro3966-sup-0006-Fig6.tiff]

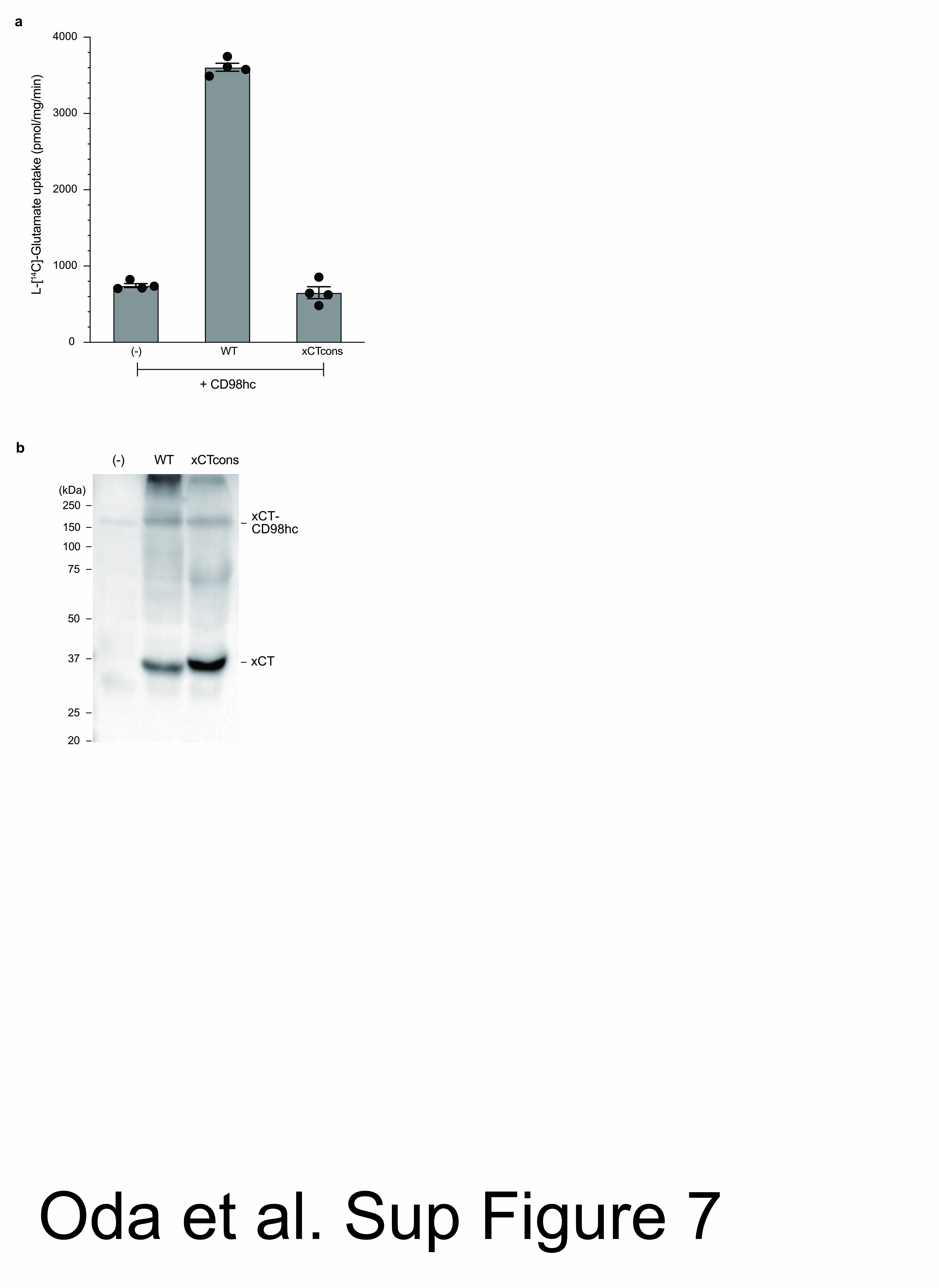

Supplement: Supplementary file 1 — Figure S1 Supporting information [file PRO-29-2398-s001.zip › pro3966-sup-0007-Fig7.tiff]
